# Current strategies of cerebral oxygenation monitoring in general intensive care units: a narrative review

**DOI:** 10.62838/jccm-2026-0030

**Published:** 2026-10-30

**Authors:** Fatos Sada, Rudin Domi, Gentian Huti, Asead Abdyli, Filadelfo Coniglione, Daniele Guerino Biasucci, Anne-Marie Camilleri-Podesta, Joana Berger-Estilita, Marius Novac

**Affiliations:** Department of Anesthesiology and Reanimation, Faculty of Medicine, University of Pristina,Kosovo; University of Medicine, Tirana, Albania; American Hospital 3, Tirana, Albania; Tor Vergata' University of Rome, Rome, Italy; Mater Dei Hospital, Msida, Malta; University of Bern, Bern, Switzerland; University of Medicine and Pharmacy, Craiova, Romania

**Keywords:** cerebral oxygenation, brain tissue oxygen tension, jugular bulb saturation, near infrared spectroscopy, multimodal neuromonitoring, intracranial pressure, transcranial doppler, microdialysis

## Abstract

**Introduction:**

Cerebral hypoxia represents the final metabolic consequence of sustained intracranial pressure and impaired autoregulation. Continuous cerebral oxygen monitoring is essential to prevent secondary ischemic injury in neurocritical care particularly in severe traumatic brain injury, malignant infarction, and aneurysmal subarachnoid hemorrhage. It also guides management of systemic disturbances such as shock, sepsis, and respiratory failure and is increasingly used perioperatively in high-risk cardiac, carotid, and neurosurgical procedures.

**Aim of the study:**

To synthesize physiological principles, monitoring modalities, and clinical applications of cerebral oxygenation across neurocritical care and perioperative settings, emphasizing bedside interpretation and integration within multimodal neuromonitoring.

**Materials and methods:**

PubMed and Scopus were searched (January 2010–January 2025) using MeSH terms and keywords including “cerebral oxygenation,” “brain tissue oxygen tension,” “jugular bulb saturation,” and “near-infrared spectroscopy.” Fifty-eight English-language studies (randomized controlled trials, observational studies, reviews, and editorials) were included. Non-English publications, animal studies, conference abstracts, unavailable full texts, and non–peer-reviewed articles were excluded.

**Results:**

Brain tissue oxygen tension, jugular bulb oximetry, and near-infrared spectroscopy provide complementary insights. Brain tissue oxygen tension is invasive and region-specific (<25 mmHg indicates high risk), jugular bulb oximetry reflects global cerebral oxygen extraction, and near-infrared spectroscopy enables noninvasive regional trend monitoring. Multimodal neuromonitoring integrating these with intracranial pressure, transcranial Doppler, and electroencephalography improves detection and management of secondary ischemia. In neurocritical care, invasive monitoring supports targeted interventions, whereas in ICU and perioperative settings, multimodal strategies balance diagnostic yield and feasibility.

**Conclusions:**

Cerebral oxygen monitoring should be routinely implemented in high-risk patients. Multimodal neuromonitoring should be prioritized to detect hypoxia early and guide individualized cerebral perfusion strategies. Near-infrared spectroscopy should be used for continuous monitoring, while invasive modalities should be reserved for high-risk neurocritical patients. Standardized thresholds and protocolized approaches should be established through prospective, outcome-driven research.

## Introduction

Cerebral hypoxia is the final metabolic consequence of sustained intracranial pressure (ICP) elevation and impaired cerebral perfusion. When cerebral perfusion pressure falls below a critical threshold, oxygen delivery becomes insufficient, leading to metabolic disturbances and potential irreversible neuronal injury [1]. Preventing secondary ischemic brain damage remains a central objective in patients with acute neurological injury and severe systemic critical illness.

Several techniques have been developed to monitor cerebral oxygenation and are widely used in neurocritical care, particularly in traumatic brain injury, malignant infarction, and aneurysmal subarachnoid hemorrhage. Their application is expanding to general intensive care and perioperative medicine, where systemic insults such as shock, sepsis, respiratory failure, or hypotension may disrupt cerebral oxygen balance [2].

However, the clinical value of cerebral oxygen monitoring remains incompletely defined, with heterogeneity in modalities, thresholds, and patient populations. This narrative review summarizes current evidence on monitoring techniques, their physiological basis, clinical applications, and limitations, highlighting ongoing controversies and knowledge gaps relevant to multi-modal neuromonitoring.

Beyond summarizing the literature, this article proposes a physiology-based framework integrating systemic determinants of oxygen delivery with regional and global cerebral oxygenation monitoring modalities. It highlights the complementary roles and limitations of PbtO_2_, SjvO_2_, and near-infrared spectroscopy across neurocritical, general intensive care, and perioperative settings, and proposes a practical multimodal monitoring algorithm to support bedside interpretation and individualized patient management.

A structured literature search of PubMed and Scopus using predefined keywords and inclusion criteria was conducted to identify relevant studies, as detailed in the following Methods section.

## Methods

### Design

This narrative review was designed to provide an integrative overview of cerebral oxygenation monitoring across neurocritical care, intensive care, and perioperative settings. Given the heterogeneity of monitoring modalities, study designs, and clinical contexts, a narrative approach was selected to allow synthesis of physiological principles, technical aspects, and clinical applications that are not amenable to formal systematic analysis or meta-analysis.

A targeted literature search was conducted in the PubMed and Scopus databases to identify relevant publications on cerebral oxygenation monitoring in neurosurgery and neurocritical care. The search covered articles published between January 2010 and January 2025 and combined Medical Subject Headings (MeSH) and free-text terms related to cerebral oxygenation and multimodal neuromonitoring. Key search terms included “cerebral oxygenation,” “brain tissue oxygen tension,” “jugular bulb saturation,” “near-infrared spectroscopy,” “intracranial pressure,” “multi-modal neuromonitoring,” “subarachnoid hemorrhage,” “traumatic brain injury,” “hypotension,” and “sepsis,” used individually and in relevant combinations.

The review focused on human studies published in English, including randomized controlled trials, observational studies, and both narrative and systematic reviews. Editorials and expert commentaries were included when they provided relevant conceptual or clinical insights. Studies were screened by the authors based on relevance to physiological mechanisms, monitoring techniques, and clinical applicability. Disagreements regarding inclusion or interpretation were resolved through discussion until consensus was reached. Fifty-eight of the 98 papers were eligible for inclusion in this review (Figure 1).

**Fig. 1.**
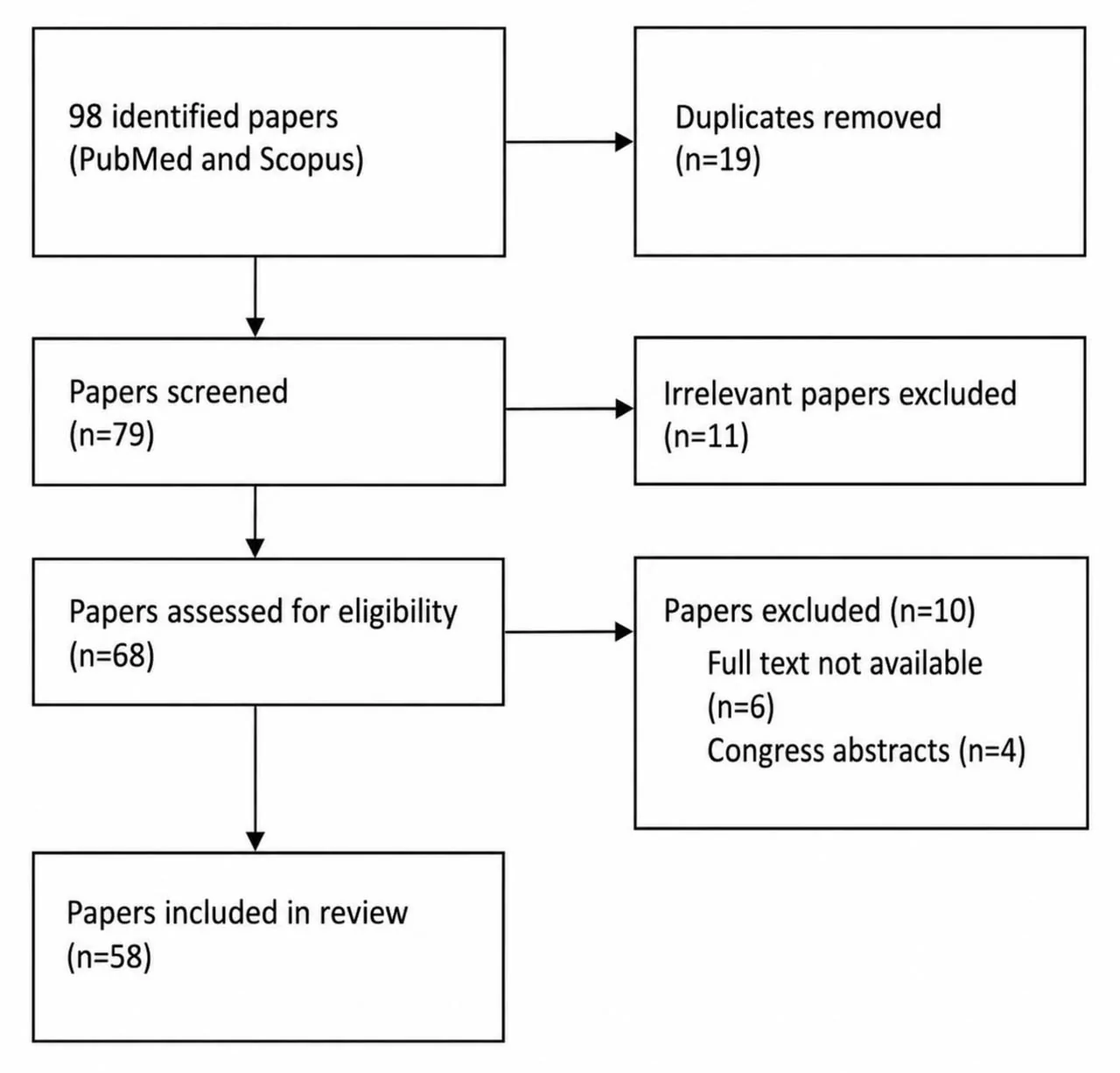
Inclusion criteria diagram.

Included studies were appraised qualitatively using predefined criteria to enhance transparency, including study design, sample size and population characteristics, methodological rigor, risk of bias (selection, measurement, confounding), and consistency of findings with established physiological principles. Greater interpretive weight was assigned to higher-quality evidence (e.g., randomized and prospective studies), while lower-quality or heterogeneous data were used to contextualize emerging concepts and areas of uncertainty.

The selected literature was synthesized narratively to provide an overview of current evidence, ongoing controversies, and the clinical relevance of cerebral oxygenation monitoring. As this was a literature-based review, informed consent was not required.

## Results and discussion

### 1. Cerebral oxygenation monitoring (physiology and monitoring techniques)

Cerebral oxygenation is determined by a complex interplay of systemic and intracranial factors. Key determinants include cardiac output, mean arterial pressure, partial arterial oxygen (PaO_2_), and carbon dioxide pressures (PaCO_2_), hemoglobin concentration, and intracranial pathophysiology such as elevated ICP, vasospasm, ischemic lesions, or massive intracranial hemorrhage. For each of these factors, specific monitoring modalities have been developed to provide reliable information on cerebral oxygenation and to guide targeted therapeutic strategies. Several of these methods have been incorporated into routine clinical practice. Brain oxygenation monitoring can be performed using several techniques, including measurement of brain tissue oxygen tension (PbtO_2_), jugular bulb oximetry (SjvO_2_), and near-infrared spectroscopy (NIRS) [3]. Each method provides valuable but distinct information regarding cerebral oxygen delivery and utilization. PbtO_2_ monitoring offers a direct assessment of local tissue oxygen levels within a specific region of the brain, reflecting the balance between cerebral blood flow, oxygen delivery, and metabolic demand [4]. It is particularly useful in detecting regional hypoxia that may not be apparent through global monitoring parameters. Jugular bulb oximetry, in contrast, provides a global estimate of cerebral oxygen extraction by measuring the oxygen saturation of venous blood draining from the brain. It is helpful for identifying global cerebral hypoxia or hyperoxia, but it lacks regional specificity and may be influenced by extracerebral contamination or improper catheter positioning. Near-infrared spectroscopy is a non-invasive method that estimates regional cerebral oxygen saturation through the absorption characteristics of oxygenated and deoxygenated hemoglobin. It allows continuous bedside monitoring and trend analysis, although its accuracy can be affected by extracerebral tissue interference and variations in scalp thickness or skin pigmentation. Together, these techniques complement each other and can guide targeted interventions aimed at optimizing cerebral oxygenation and preventing secondary brain injury.

### 2. Brain tissue oxygen tension (evidence and limitations)

Even when intracranial pressure (ICP) and cerebral perfusion pressure (CPP) are managed according to current guidelines, episodes of cerebral tissue hypoxia may still occur. These may arise from microvascular dysfunction, impaired oxygen diffusion, mitochondrial failure, or systemic disturbances that limit oxygen delivery despite apparently adequate global physiological parameters. Observational studies have consistently linked reduced PbtO_2_ with worse neurological outcomes after severe traumatic brain injury (TBI), including delayed recovery, cognitive impairment, and long-term disability. However, these associations remain observational and do not demonstrate that correcting PbtO_2_ abnormalities improves outcomes. Interest in PbtO_2_ monitoring reflects the recognition that ICP and CPP alone may incompletely capture cerebral oxygenation following brain injury.

PbtO_2_ is measured using invasive probes inserted into the brain parenchyma, allowing continuous assessment of regional oxygen availability. This approach can detect focal hypoxic episodes not reflected by global parameters. However, important limitations exist. Measurements represent a small tissue volume and may not reflect global cerebral physiology, while probe placement typically near the contusion or in apparently normal tissue, can influence the recorded values. The invasive nature of the technique carries a small risk of hemorrhage or tissue injury and requires a stabilization period before reliable measurements are obtained [5].

Observational evidence supports the clinical relevance of low PbtO_2_ levels. Wettervik et al. reported that sustained reductions, particularly below 25 mmHg and more markedly below 15 mmHg, were associated with unfavorable neurological outcomes [6]. These associations may be confounded by injury severity or systemic disturbances and are strongest when occurring along-side other physiological abnormalities, suggesting that tissue hypoxia reflects broader cerebral metabolic distress rather than an isolated therapeutic target.

Randomized trials have evaluated whether PbtO_2_-guided therapy improves outcomes. In the BOOST II trial, Okonkwo et al. randomized 119 patients across ten intensive care units to management guided by ICP alone or by combined ICP and PbtO_2_ monitoring [7]. Combined monitoring significantly reduced the burden of cerebral hypoxia, demonstrating physiological efficacy, but the trial was not powered to detect clinical outcome differences. In contrast, the multicenter OXY-TC trial conducted by Payen et al. found no significant improvement in six-month functional outcomes with combined monitoring [8]. Differences in study design, including intervention strategies and patient heterogeneity, may partly explain these findings. Post-hoc analyses suggested possible benefit among selected patients, indicating that patient selection may influence clinical utility.

Two large ongoing randomized trials aim to clarify these uncertainties. The BOOST-3 trial is enrolling more than 1,000 patients with severe TBI to compare ICP-guided management with combined ICP and PbtO_2_-guided therapy [9,10]. Similarly, the BONAN-ZA-GT trial is evaluating early PbtO_2_-guided treatment versus standard ICP/CPP-based care in 860 patients [11,12]. Their results will be critical in determining whether physiological improvements translate into meaningful clinical benefit.

Meta-analytic evidence remains inconclusive. A systematic review by Chang et al. reported a modest increase in favorable functional outcomes with combined monitoring but no reduction in mortality [13]. However, small sample sizes and variability in monitoring protocols limit firm conclusions. Observational cohorts, such as Narotam et al. [14], have reported improved outcomes with PbtO_2_-directed protocols, although these findings are limited by confounding.

Overall, PbtO_2_ monitoring provides valuable physiological information and can identify regional hypoxia not detected by ICP and CPP alone. PbtO_2_ monitoring is best regarded as a complementary physiological tool that enhances detection of cerebral hypoxia, with clinical benefit dependent on its integration into targeted, individualized management rather than use as a stand-alone therapeutic target.

### 3. Jugular bulb oximetry (evidence and limitations)

Jugular bulb oximetry is a monitoring modality that reflects the global balance between cerebral oxygen delivery and consumption rather than regional brain oxygenation. A catheter positioned in the jugular bulb measures oxygen saturation in cerebral venous blood, either intermittently or continuously, providing an indirect estimate of overall cerebral oxygen extraction. Unlike PbtO_2_ monitoring, which reflects localized oxygenation, SjvO_2_ represents averaged venous out-flow from the entire brain. Consequently, its clinical value lies primarily in conditions associated with diffuse cerebral injury, such as severe TBI, subarachnoid hemorrhage, and selected neurosurgical procedures where trends in global oxygen extraction may help identify cerebral hypoperfusion or increased metabolic demand [15,16,17]. However, because it reflects global rather than regional oxygenation, SjvO_2_ may fail to detect focal ischemia in heterogeneous brain injuries. In addition, its invasive nature, technical complexity, and the availability of more region-specific techniques have reduced its routine use in modern neurocritical care.

Physiologically, low SjvO_2_ values indicate increased cerebral oxygen extraction and may suggest cerebral ischemia. Several studies support its potential prognostic and diagnostic value. Senapathi et al. observed that SjvO_2_ trends during the first 72 hours after severe TBI correlated with mortality, suggesting that sustained abnormalities may reflect ongoing cerebral metabolic distress [18]. Similarly, Moritz et al. reported that jugular bulb monitoring during carotid surgery could detect cerebral ischemia during carotid clamping with reasonable sensitivity and specificity [19], supporting its role in identifying global perfusion imbalance.

However, other investigations highlight important limitations. Lee et al. found that SjvO_2_ measurements did not reliably correlate with cerebral blood flow or metabolic markers in post–cardiac arrest patients [20]. Favier et al. suggested that in cases of elevated SjvO_2_ in comatose cardiac arrest survivors, EEG may help identify patients likely to benefit from hypertonic saline therapy [21]. Likewise, Keller et al. reported poor correspondence between SjvO_2_ changes and cerebral blood flow in hemispheric stroke [22]. Chan et al. found that SjvO_2_ below 45% was associated with significant metabolic derangements and may represent an ischemic threshold [23]. In contrast, Richter et al. observed that low SjvO_2_ episodes were rare after cardiac arrest and that higher values were paradoxically associated with worse outcomes, likely reflecting impaired oxygen utilization [24]. These findings illustrate a key limitation: as a global measure, SjvO_2_ may not detect regional ischemia or localized metabolic failure.

Additional studies emphasize the importance of physiological context. Ramos et al. demonstrated that head elevation reduced intracranial pressure without affecting SjvO_2_, indicating preserved global oxygen balance despite changes in intracranial dynamics [25]. Similarly, Kumari et al. reported comparable cerebral oxygenation during neurosurgery despite differences in SjvO_2_ values with transfusion strategies [26]. These findings suggest that SjvO_2_ fluctuations may reflect systemic or procedural factors rather than direct changes in cerebral perfusion.

Given these limitations, multimodal approach has been proposed to enhance interpretation. Tsaousi et al. suggested that combining SjvO_2_ with NIRS and transcranial Doppler provides complementary insights into cerebral perfusion and oxygenation [27]. Similarly, Ansar et al. highlighted that SjvO_2_ and NIRS assess different physiological domains, with SjvO_2_ reflecting global balance and NIRS providing regional cortical trends [28].

More broadly, reviews have reached similar conclusions. Oddo et al. reported that while invasive techniques such as PbtO_2_ better detect regional hypoxia and support individualized strategies, SjvO_2_ has lower diagnostic precision and limited evidence supporting routine use [29]. Consequently, it is increasingly considered a complementary physiological indicator rather than a stand-alone modality.

Table 1. summarizes key studies designs on SjvO2 use for cerebral oxygenation monitoring.

**Table 1:** Summary of Studies on Jugular Bulb Oximetry Monitoring.

**Study (Author, Year)**	**Population / Design**	**Monitoring Method**	**Conclusions / Implications**
**Ramos et al.**	Systematic review & meta-analysis; 16 studies on head elevation in acute brain injury	ICP, CPP, SjvO_2_, PbtO_2_, AVDO_2_	Moderate head elevation (30°) effectively lowers ICP without impairing perfusion or oxygenation.
**Tsaousi et al.**	Review on perioperative cerebral desaturation	NIRS, SjvO_2_, TCD (multimodal)	Multimodal monitoring improves detection but no proven outcome benefit yet.
**Senapathi et al.**	63 patients with severe TBI, observational	SjvO_2_	SjvO_2_ is a prognostic indicator of mortality in severe TBI.
**Moritz et al.**	48 patients undergoing carotid surgery (regional anesthesia)	SjvO_2_	SjvO_2_ effectively detects cerebral hypoperfusion during carotid clamping.
**Favier et al.**	Retrospective cohort, comatose cardiac arrest survivors	SjvO_2_ + EEG	Elevated SjvO_2_ may indicate perivascular edema; EEG can guide HTS therapy benefit.
**Kumari et al.**	80 adults in elective neuro-oncological surgery, RCT	SjvO2	Transfusion of older PRBCs (>14 days) appears safe in neuro-oncologic surgery.
**Lee et al.**	Retrospective analysis of 36 early post–cardiac arrest datasets	Jugular bulb parameters (%CBF, lactate/creatine ratio)	SjvO_2_ and related metrics have limited value post–cardiac arrest.
**Chan et al**.	25 severe head injury patients	SjvO_2_ + cerebral microdialysis	SjvO_2_ <45% marks onset of cerebral ischemia; represents ischemic threshold.
**Keller et al.**	10 patients with severe hemispheric stroke	Jugular bulb oximetry, ICP, CBF	SjvO_2_ unreliable for perfusion monitoring in ischemic stroke; TBI thresholds not applicable.
**Richter et al.**	Prospective study, 40 post–cardiac arrest patients	Jugular bulb oximetry	SjvO_2_ seldom detects hypoxia post–cardiac arrest; limited utility for management.
**Ansar et al.**	Systematic review	Jugular bulb oximetry, NIRS	SjvO2 is useful for global cerebral ischemia, NIRS is practical and detects focal ischemia, combined multimodal may be optimal

SjvO_2_ is best interpreted as a complementary global indicator of cerebral oxygenation in diffuse brain injury or generalized hypoperfusion and requires integration with regional monitoring modalities to guide clinical decision-making and improve diagnostic accuracy.

### 4. Near-infrared spectroscopy (evidence and limitations)

NIRS provides an indirect estimate of cerebral oxygen saturation and is widely used as a noninvasive adjunct for detecting cerebral hypoxia and guiding individualized therapeutic interventions. Its ease of application and ability to provide continuous monitoring allow its use not only in neurosurgical and neurocritical care settings but also in broader clinical contexts where cerebral oxygenation monitoring is relevant [30,31]. However, its clinical value remains debated due to methodological limitations and variability in monitoring techniques, diagnostic thresholds, and study end-points.

Viderman et al. conducted a systematic review including 2,291 patients across 19 studies and suggested that NIRS may serve as a screening tool for identifying intracranial hemorrhage in TBI, particularly in prehospital environments [32]. A negative NIRS result may help exclude hemorrhage and reduce unnecessary CT imaging, while early assessment may facilitate triage to neurosurgical centers. However, heterogeneity in devices, patient populations, and thresholds limits the strength and generalizability of these findings.

Roldan et al. highlighted the potential value of NIRS as a bedside monitoring modality during acute TBI [33]. Continuous monitoring of regional cerebral oxygen saturation may enable early detection of hypoxia and impaired perfusion, while dynamic changes may provide indirect insight into cerebrovascular autoregulation. However, most studies are observational and focus on physiological variables rather than patient-centered outcomes, limiting conclusions regarding clinical impact.

Beyond neurocritical care, NIRS has been increasingly evaluated in cardiothoracic anesthesia [34]. During cardiac surgery, cerebral oxygen saturation may fluctuate due to hemodynamic instability, arrhythmias, cardiopulmonary bypass, and circulatory arrest. In these settings, NIRS may allow early detection of cerebral desaturation and support timely corrective interventions. However, evidence for improved neurological outcomes remains inconsistent, as most studies rely on surrogate physiological endpoints.

This discrepancy is illustrated by the randomized trial by Bieze et al. in elderly patients undergoing major non-cardiac surgery [35]. Although NIRS-guided anesthesia reduced the duration of cerebral desaturation episodes, no significant reductions in postoperative morbidity or complications were observed, suggesting that detected desaturation does not always correspond to clinically relevant ischemic injury.

Several studies highlight important technical and physiological limitations of NIRS. Lukaszewski et al. reported persistently high NIRS values during brain death evaluation in a patient with massive intracranial hemorrhage, likely due to extracranial signal contamination [36]. Similarly, Pedersen et al. demonstrated that oxygen saturation values measured at the dura did not differ significantly from those recorded at the skin, indicating a substantial extracranial contribution to the signal [37]. These findings reduce the specificity of NIRS for assessing true cerebral oxygenation.

Further evidence of both sensitivity and interpretative limitations was provided by Tobias, who observed significant variability in cerebral oxygenation during one-lung ventilation despite normal systemic oxygen saturation [38]. This suggests that cerebral hypoxia may occur independently of systemic oxygenation, although the clinical significance of transient desaturation remains uncertain.

The role of NIRS in guiding transfusion strategies has also been explored. Leal-Noval et al. reported that a NIRS-guided transfusion protocol resulted in fewer transfusions compared with a hemoglobin-based strategy, although no clear improvement in major clinical outcomes was observed [39].

Several studies suggest that NIRS may assist in maintaining cerebral oxygen balance, guiding transfusion decisions, identifying intracranial hemorrhage, and detecting intracranial hypertension [40,41]. However, variability in study design, monitoring devices, and intervention protocols limits direct comparison between studies.

The technical limitations of NIRS are well summarized by Moerman et al. [42]. Because oxygen saturation is estimated indirectly through light absorption, measurements are susceptible to interference from ambient light, extracranial tissues, and variability in tissue composition and probe positioning. Consequently, NIRS is more useful for monitoring trends rather than absolute values. In addition, it reflects a mixed vascular compartment and evaluates only a limited cortical region, meaning that focal ischemia outside the monitored area may remain undetected.

NIRS allows continuous, noninvasive monitoring of regional cerebral oxygenation and may detect subclinical hypoxia even when systemic oxygenation appears normal [30,31,32,33,34,35,36,37,38,39,40,41,42]. However, methodological limitations, extracranial signal contamination, and lack of standardized thresholds complicate interpretation. Table 2 summarizes monitoring techniques and their clinical use.

**Table 2:** Cerebral Oxygen Monitoring Techniques.

**Technique**	**Description**	**Clinical Availability / Typical Use**
**Jugular bulb venous oximetry**	Provides a global estimate of cerebral oxygen balance but is invasive and insensitive to focal ischemia.	Continuous monitoring of global cerebral oxygenation in severe TBI patients, rare use in SAH, neurosurgery, cardiac surgery
**Brain tissue oxygen tension monitoring**	Offers direct regional measurement but requires neurosurgical probe placement.	Widely used in neurocritical care and research settings, particularly in severe TBI, SAH, and selected neurocritical patients
**Transcranial Doppler ultrasonography**	Non-invasive and portable but operator-dependent, providing only indirect oxygenation data.	Specialized centers; neurocritical care, stroke, SAH, perioperative monitoring
**Positron emission tomography (PET)**	The gold standard for assessing cerebral oxygen metabolism but impractical for continuous use.	Primarily research use; limited clinical availability
**Functional magnetic resonance imaging (fMRI)**	Offers high spatial resolution but is non-continuous and unsuitable for unstable patients.	Research and elective clinical applications
**Near-infrared spectroscopy (NIRS)**	Allows continuous, non-invasive monitoring but is limited by extracerebral signal contamination and lack of standardized thresholds.	Widely available; used in cardiac, thoracic, vascular surgery, ICU, and prehospital screening

NIRS is a practical and widely applicable tool for continuous monitoring and trend detection, but its limitations require cautious interpretation and integration with other neuromonitoring modalities to inform clinical decision-making.

### 5. Multimodal neuromonitoring concepts (evidence and limitations)

For many years, therapeutic strategies, follow-up, and prognostic evaluation in neurocritical care relied primarily on clinical neurological examination and neuroimaging. While essential, this approach provides limited insight into secondary cerebral injury related to impaired oxygenation and perfusion, thereby over-looking dynamic pathophysiological processes that evolve after the primary insult. Multimodal neuromonitoring (MMM) has emerged to address these limitations by integrating multiple monitoring tools tailored to evolving cerebral physiology. The principal aims of MMM are early detection and characterization of secondary brain injury, continuous monitoring in patients with a Glasgow Coma Scale (GCS) score < 9 due to traumatic brain injury or subarachnoid hemorrhage, and guidance of timely, targeted interventions to limit secondary damage.

MMM combines clinical assessment, neuroimaging, and both invasive and non-invasive monitoring modalities, enabling a more accurate and individualized evaluation of cerebral physiology and prognosis. Core components include intracranial pressure monitoring via intraparenchymal or intraventricular catheters; optimization of systemic hemodynamics to maintain CPP around 60–70 mmHg; assessment of cerebral blood flow, most commonly with transcranial Doppler ultrasonography; evaluation of cerebral oxygenation using NIRS, PbtO_2_, and SjvO_2_; and analysis of cerebral metabolism via microdialysis [43,44,45]. Table 3 summarizes key MMM concepts.

**Table 3:** Key concepts of Multimodal Neuromonitoring [43].

**Physiological concept**	**Parameter**	**Monitoring tool**	**Clinical importance**
**ICP**	ICP	Intraparenchymal/intraventricular catheter	Cerebral edema, herniation
**Cerebral blood flow (CBF)**	Blood velocity	Transcranial doppler	Optimizing cerebral perfusion, vasospasm
**Cerebral autoregulation**	Pressure reactivity (PRx)	Transcranial doppler	Optimizing cerebral perfusion
**Cerebral oxygenation**	PbtO2, SjvO2, NIRS	Intraparenchymal catheter, fiberoptic catheter in internal jugular vein and positioned in jugular bulb, frontal region electrodes	Global cerebral ischemia (SjvO2), focal cerebral ischemia (PbtO2, NIRS)
**Cerebral metabolism**	Lactate/Piruvate (L/P) ratio	Probe with semipermeable membrane	Cerebral ischemia, anaerobic metabolism

Despite increasing adoption, MMM approaches vary across studies, limiting direct comparison between strategies [46]. In a review by Taş et al., 45 studies were analyzed, of which only ten specifically evaluated MMM-guided therapy. Intracranial monitoring and PbtO_2_ were the most frequently used modalities, while SjvO_2_ use declined with increased adoption of NIRS. Importantly, studies incorporating MMM were associated with improved patient outcomes [47].

By integrating data on autoregulation, blood pressure, cerebral blood flow, oxygenation, and metabolism, MMM provides a more comprehensive understanding of cerebral physiology than conventional monitoring alone. However, further validation is needed to consolidate its clinical impact [48]. Each modality has inherent limitations, as outlined by Sharma et al. [49]. Neurological examination is valuable but requires repeated assessments; pupillometry is influenced by anatomical and physiological factors; transcranial Doppler is operator-dependent and limited to selected vascular territories; NIRS primarily reflects frontal cortical oxygenation; and PbtO_2_ provides regional information and is invasive [49].

MMM has been most extensively studied in traumatic brain injury. Casault et al. highlighted the value of components such as PbtO_2_, pressure reactivity index, and cerebral microdialysis in guiding management of severe TBI [50]. Similarly, Robba et al. demonstrated that combined ICP and PbtO_2_ monitoring supports individualized, physiology-driven management, facilitates early detection of secondary injury, and contributes to improved outcomes [51].

Its application is also expanding in subarachnoid hemorrhage, where delayed cerebral ischemia is a major determinant of outcome. A systematic review by Veldeman et al., including 47 studies, supported the role of MMM in improving outcomes related to delayed cerebral ischemia [52].

MMM has additionally been applied in the management of neurological complications in COVID-19. Battaglini et al. reported that non-invasive MMM techniques, including transcranial Doppler, optic nerve sheath diameter, and automated pupillometry, were useful for monitoring neurological complications in ICU patients [53].

Although invasive ICP monitoring remains the gold standard [54], non-invasive approaches are increasingly explored. In a prospective study of ICU patients with TBI, subarachnoid hemorrhage, or intracerebral hemorrhage, combined non-invasive measures including optic nerve sheath diameter, transcranial Doppler pulsatility index, estimated ICP, and automated pupillometry, demonstrated acceptable accuracy for detecting intracranial hypertension, with best performance achieved through combined modalities [55].

Several consensus statements have aimed to standardize MMM. The 2014 international consensus recommended monitoring ICP and CPP (moderate-quality evidence), as well as PbtO_2_ and/or SjvO_2_ (low-quality evidence), and strongly endorsed transcranial Doppler based on high-quality evidence [56]. Pressure reactivity index received a weaker recommendation, while cerebral microdialysis was strongly supported despite limited evidence [56].

More recently, Foreman et al. emphasized that both invasive (ICP, CPP, PbtO_2_) and non-invasive techniques (transcranial Doppler, NIRS, pupillometry) are essential components of MMM and should be integrated to guide individualized management [57]. Similarly, the Neurocore-iMMM Delphi consensus by Barrit et al. established standardized reporting frameworks and core outcome sets to improve consistency and comparability across studies [58]. Figure 2 integrates these concepts, linking systemic physiological variables with cerebral oxygenation monitoring.

**Fig. 2.**
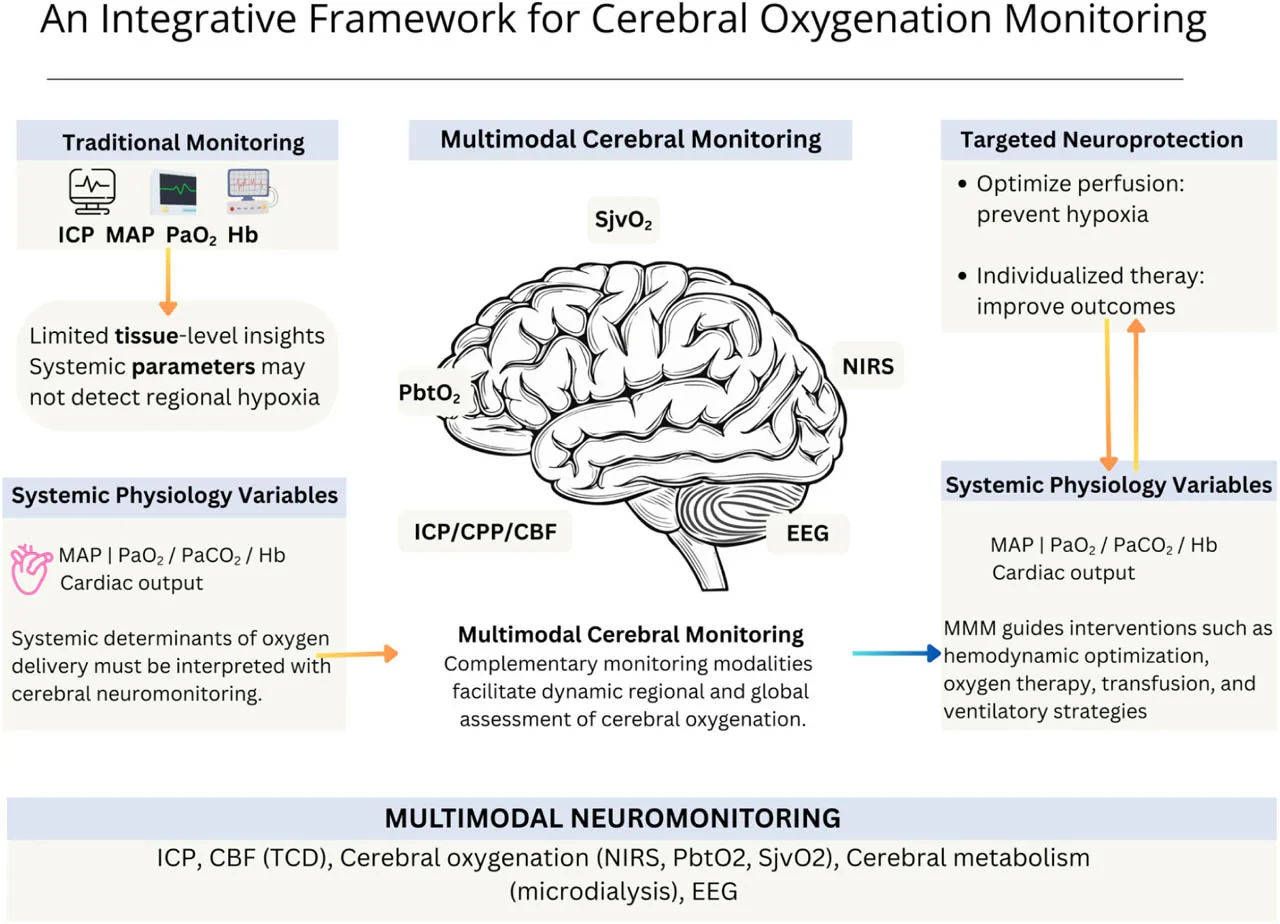
The conceptual framework linking systemic physiological determinants of oxygen delivery with cerebral monitoring modalities.

MMM provides the most comprehensive framework for assessing cerebral physiology by integrating complementary monitoring modalities, enabling individualized management while highlighting the need for further standardization and outcome-driven validation.

### 6. Clinical integration and practical recommendations of PbtO2, SjvO2, NIRS as part of MMM

PbtO_2_ monitoring is primarily suited for focal assessment of cerebral oxygenation in patients with severe traumatic brain injury and subarachnoid hemorrhage, particularly within specialized neurocritical care units; however, its inherently regional nature limits its applicability in broader intensive care settings and in cardiac surgery. In contrast, jugular bulb oximetry provides an index of global cerebral oxygen supply–demand balance and is therefore less informative in conditions characterized by focal pathology, such as hemispheric ischemic stroke. Near-infrared spectroscopy is most employed in cardiac surgery and perioperative neuro-surgical settings, where its non-invasive, continuous nature allows effective trend monitoring of cerebral oxygenation. MMM represents the reference strategy in complex neurocritical care, especially in severe traumatic brain injury and high-grade subarachnoid hemorrhage, as the integration of complementary physiological signals enables more precise and individualized patient management (table 4). Figure 3. illustrates a stepwise algorithm for cerebral oxygenation monitoring accordingly to the specific clinical contexts and monitoring objectives. Figure 4 summarizes the clinical interpretation of SjvO_2_, PbtO_2_, and NIRS thresholds, both individually and in combination to guide targeted therapy.

**Table 4:** Invasive and Non-invasive Techniques for Monitoring Cerebral Oxygenation.

**Modality**	**TBI**	**SAH**	**Hemispheric Stroke**	**Cardiac Surgery**	**Neurosurgery**	**General ICU**
**PbtO_2_ (Brain tissue oxygen tension)**	✓	✓	±	x	±	±
**SjvO_2_ (Jugular bulb oximetry)**	±	±	x	±	±	x
**NIRS (Near-infrared spectroscopy)**	±	±	±	✓	✓	±
**Multimodal neuromonitoring (ICP, CPP, PbtO_2_, microdialysis, EEG, etc.)**	✓	✓	±	x	±	±

**Fig. 3.**
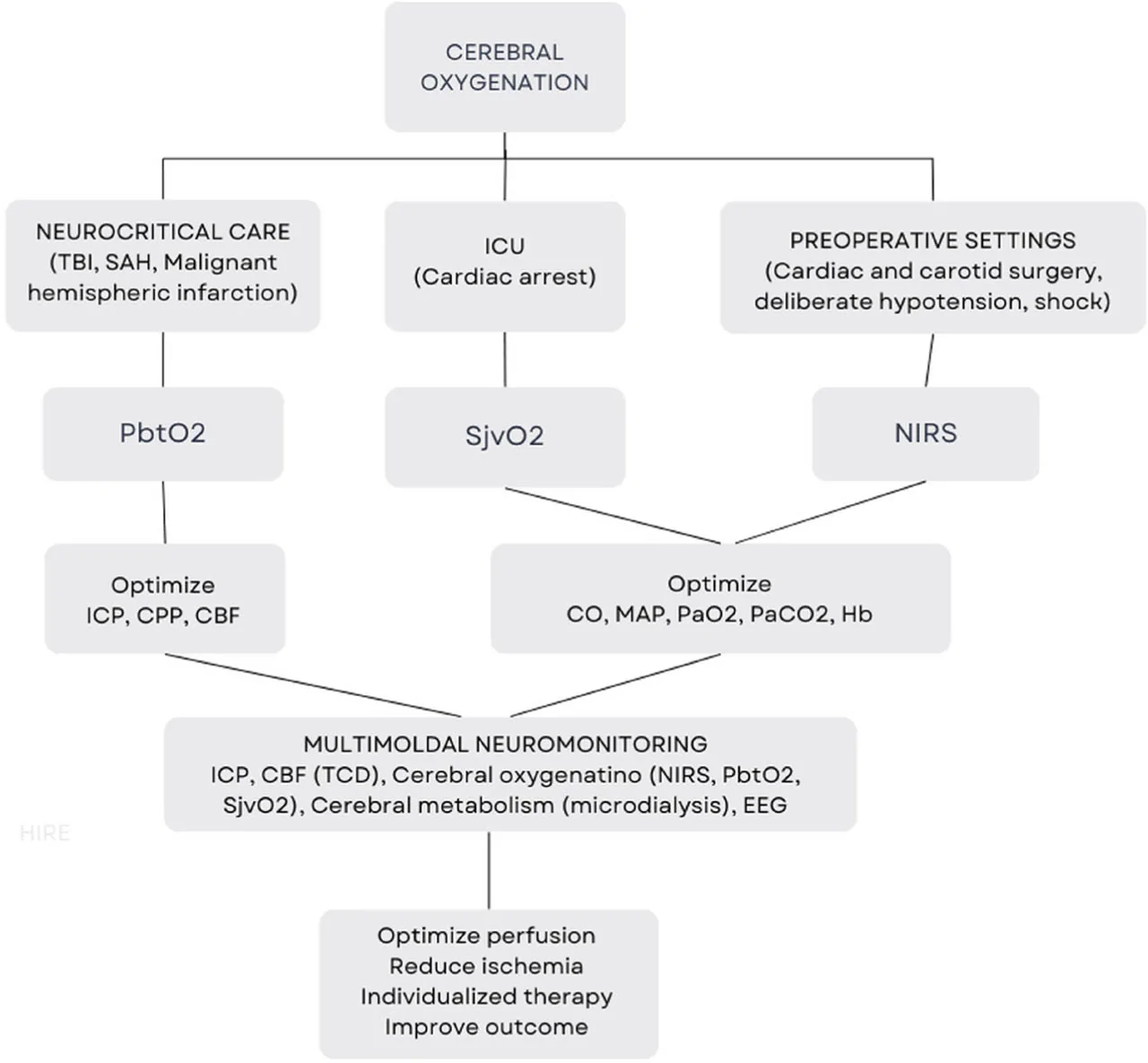
Clinical algorithm for multimodal cerebral oxygenation monitoring incorporating NIRS, SjvO_2_, and PbtO_2_, adapted to varying clinical scenarios and monitoring goals.

**Fig. 4.**
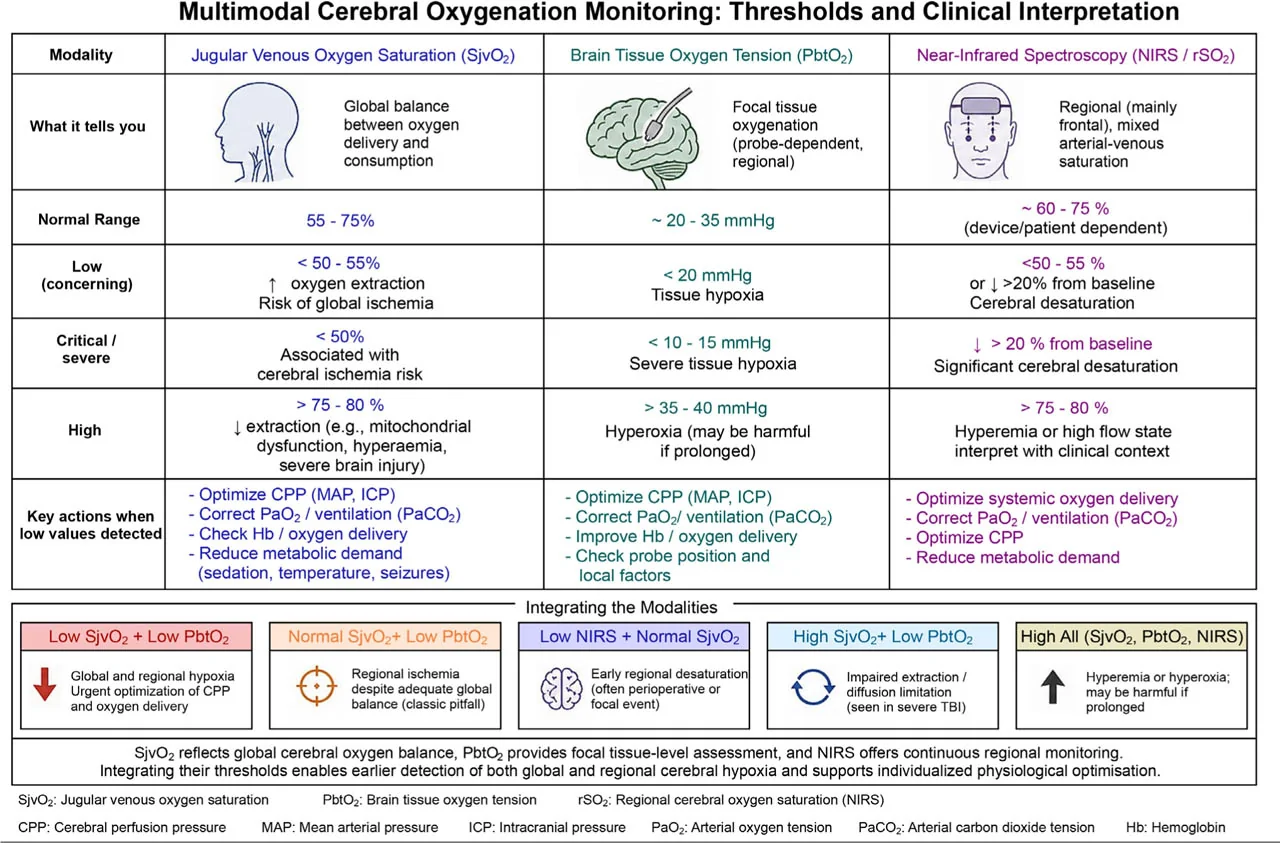
Clinical interpretation of SjvO2, PbtO2, and NIRS thresholds.

### 7. Limitations

This review has several inherent limitations. As a narrative review, it was intended to provide an integrative overview rather than a formal quantitative synthesis; although a structured search strategy was applied, some relevant studies may not have been identified. The literature spans diverse patient populations, monitoring techniques, clinical settings, and outcome measures, which constrains direct comparability but reflects the complexity of cerebral oxygenation monitoring in clinical practice. Much of the current evidence particularly for PbtO_2_ and SjvO_2_-guided management, derives from observational studies and smaller randomized trials and therefore does not yet establish definitive effects on patient-centered outcomes. In addition, restriction to English-language publications may have excluded some relevant data. Despite these limitations, the evidence supports a coherent physiological and clinical rationale for integrating cerebral oxygenation monitoring into multimodal strategies. This review provides a structured framework for clinical interpretation and identifies key priorities for future research to standardize approaches and clarify their impact on meaningful neurological outcomes.

### 8. Future directions

Future research should focus on defining clinically meaningful thresholds for intervention, clarifying which patient populations benefit most from specific monitoring strategies, and determining whether protocol-driven management based on cerebral oxygenation improves neurological outcomes. Large prospective studies and randomized trials are needed to establish standardized monitoring algorithms and to better integrate cerebral oxygenation monitoring into evidence-based neuroprotective strategies.

## Conclusions

Continuous cerebral oxygenation monitoring has become an important component of neurocritical, intensive, and perioperative care, enabling early detection of hypoxia and more individualized management. Different modalities provide complementary clinical information. PbtO_2_ offers direct regional assessment and is most useful in severe traumatic brain injury and focal ischemia. SjvO_2_ reflects the global balance between oxygen delivery and consumption and is more informative in diffuse brain injury or systemic hypoperfusion. NIRS provides a noninvasive, continuous estimate of regional oxygenation and is particularly suited for perioperative monitoring and bedside trend assessment.

Despite these advantages, uncertainties remain. Evidence linking routine monitoring to improved long-term outcomes is still evolving, and optimal thresholds and integration strategies are not fully defined. Each modality also has limitations, PbtO_2_ is invasive and localized, SjvO_2_ lacks sensitivity to focal pathology, and NIRS is susceptible to extracranial influences, highlighting that no single technique is sufficient on its own.

Clinical use should therefore be context driven. Cerebral oxygenation data should be interpreted alongside systemic physiology and neuroimaging, with multi-modal approaches combining PbtO_2_, SjvO_2_, NIRS, and intracranial pressure monitoring providing the most comprehensive assessment. In perioperative settings, NIRS offers a practical tool for continuous surveillance and early intervention.

In summary, PbtO_2_, SjvO_2_, and NIRS each address different aspects of cerebral oxygenation, and their integration within multimodal monitoring provides the most effective strategy for detecting hypoxia and guiding individualized care.

## References

[1] Bögli SY, Olakorede I, Beqiri E (2025). Cerebral perfusion pressure targets after traumatic brain injury: a reappraisal. Crit Care..

[2] Domi R, Coniglione F, Huti G, Lilaj K (2025). Permissive strategies in intensive care units (ICUs): actual trends?. Anesth Pain Med (Seoul).

[3] Haitsma IK, Maas AI (2002). Advanced monitoring in the intensive care unit: brain tissue oxygen tension. Curr Opin Crit Care.

[4] Hawryluk GWJ, Citerio G, Hutchinson P (2022). Intracranial pressure: current perspectives on physiology and monitoring. Intensive Care Med.

[5] Lindner A, Rass V, Ianosi BA (2021). The Importance of PbtO2Probe Location for Data Interpretation in Patients with Intracerebral Hemorrhage. Neurocrit Care.

[6] Svedung Wettervik T, Beqiri E (2023). Brain tissue oxygen monitoring in traumatic brain injury-part II: isolated and combined insults in relation to outcome. Crit Care..

[7] Okonkwo DO, Shutter LA, Moore C (2017). Brain Oxygen Optimization in Severe Traumatic Brain Injury Phase-II: A Phase II Randomized Trial. Crit Care Med.

[8] Payen JF, Vilotitch A, Gauss T (2025). Sex Differences in Neurological Outcome at 6 and 12 Months Following Severe Traumatic Brain Injury. An Observational Analysis of the OXY-TC Trial. J Neurotrauma..

[9] Bernard F, Barsan W, Diaz-Arrastia R, Merck LH, Yeatts S, Shutter LA (2022). Brain Oxygen Optimization in Severe Traumatic Brain Injury (BOOST-3): a multicentre, randomised, blinded-endpoint, comparative effectiveness study of brain tissue oxygen and intracranial pressure monitoring versus intracranial pressure alone. BMJ Open..

[10] Lazaridis C, Foreman B (2023). Management Strategies Based on Multi-Modality Neuromonitoring in Severe Traumatic Brain Injury. Neurotherapeutics.

[11] Udy AA, Jeffcote T, Battistuzzo CR (2025). Brain tissue oxygen monitoring for severe traumatic brain injury: the international multicentre randomised controlled BONANZA-GT study protocol. BMJ Open..

[12] BONANZA-ANZICS [Internet] (2021). https://www.anzics.com.au/current-active-endorsed-research/bonanza/.

[13] Chang Y, Wong CE, Hsu HH (2025). Brain Tissue Oxygen Combined with Intracranial Pressure Monitoring in Patients with Severe Traumatic Brain Injury: An Updated Systematic Review and Pooled Analysis Following the OXY-TC Trial. World Neurosurg.

[14] Narotam PK, Morrison JF, Nathoo N (2009). Brain tissue oxygen monitoring in traumatic brain injury and major trauma: outcome analysis of a brain tissue oxygen-directed therapy. J Neurosurg.

[15] Zhong W, Ji Z, Sun C (2021). A Review of Monitoring Methods for Cerebral Blood Oxygen Saturation. Healthcare..

[16] White H, Baker A (2002). Continuous jugular venous oximetry in the neurointensive care unit--a brief review. Can J Anaesth.

[17] Schell RM, Cole DJ (2000). Cerebral monitoring: jugular venous oximetry. Anesth Analg.

[18] Senapathi TGA, Wiryana M, Sinardja K (2017). Jugular bulb oxygen saturation correlates with Full Outline of Responsiveness score in severe traumatic brain injury patients. Open Access Emerg Med..

[19] Moritz S, Kasprzak P, Woertgen C, Taeger K, Metz C (2008). The accuracy of jugular bulb venous monitoring in detecting cerebral ischemia in awake patients undergoing carotid endarterectomy. J Neurosurg Anesthesiol.

[20] Lee HY, Mamadjonov N, Jung YH (2025). Relationships of Jugular Bulb Parameters with Cerebral Perfusion and Metabolism After Resuscitation from Cardiac Arrest: A Post-Hoc Analysis of Experimental Studies Using a Minipig Model. Neurocrit Care.

[21] Faiver L, Coppler PJ, Tam J (2024). Association of hyperosmolar therapy with cerebral oxygen extraction after cardiac arrest. Resuscitation.

[22] Keller E, Steiner T, Fandino J, Schwab S, Hacke W (2002). Jugular venous oxygen saturation thresholds in trauma patients may not extrapolate to ischemic stroke patients: lessons from a preliminary study. J Neurosurg Anesthesiol.

[23] Chan MT, Ng SC, Lam JM, Poon WS, Gin T (2005). Re-defining the ischemic threshold for jugular venous oxygen saturation--a microdialysis study in patients with severe head injury. Acta Neurochir Suppl..

[24] Richter J, Sklienka P, Setra AE, Zahorec R, Das S, Chatterjee N (2021). Is jugular bulb oximetry monitoring associated with outcome in out of hospital cardiac arrest patients?. J Clin Monit Comput.

[25] Ramos MB, Britz JPE, Telles JPM (2024). The Effects of Head Elevation on Intracranial Pressure, Cerebral Perfusion Pressure, and Cerebral Oxygenation Among Patients with Acute Brain Injury: A Systematic Review and Meta-Analysis. Neurocrit Care.

[26] Kumari A, Singh BP, Prakash R, Hemlata H, Singh D, Kohli M (2025). Effect of Red Blood Cell Storage Duration on Cerebral Blood Oxygenation in Elective Cranial Surgery: A Prospective Randomized Controlled Study. Cureus..

[27] Tsaousi G, Tramontana A, Yamani F, Bilotta F (2021). Cerebral Perfusion and Brain Oxygen Saturation Monitoring with: Jugular Venous Oxygen Saturation, Cerebral Oximetry, and Transcranial Doppler Ultrasonography. Anesthesiol Clin.

[28] Ansar P, Mohseni G, Behnaz F, Abniki M, Shojaei S P (2025). Jugular Venous Oxygen Saturation (SjvO2) vs. Cerebral Pulse Oximetry in Traumatic Brain Injury (TBI): A Systematic Review. J Cell Mol Anesth.

[29] Oddo M, Bösel J, Participants in the International Multidisciplinary Consensus Conference on Multimodality Monitoring (2014). Monitoring of brain and systemic oxygenation in neurocritical care patients. Neurocrit Care..

[30] Robba C, Battaglini D, Rasulo F, Lobo FA, Matta B (2023). The importance of monitoring cerebral oxygenation in non-brain injured patients. J Clin Monit Comput.

[31] Davies DJ, Su Z, Clancy MT, Lucas SJ, Dehghani H, Logan A, Belli A (2015). Near-Infrared Spectroscopy in the Monitoring of Adult Traumatic Brain Injury: A Review. J Neurotrauma.

[32] Viderman D, Ayapbergenov A, Abilman N, Abdildin YG (2021). Near-infrared spectroscopy for intracranial hemorrhage detection in traumatic brain injury patients: A systematic review. Am J Emerg Med..

[33] Roldán M, Kyriacou PA (2021). Near-Infrared Spectroscopy (NIRS) in Traumatic Brain Injury (TBI). Sensors.

[34] Sarkar M, Kanchi M (2022). Cerebral oximetry using near-infrared spectroscopy (NIRS) in cardiovascular and thoracic anaesthesia: A review [Internet]. Indian J Clin Anaesth.

[35] Bieze M, Foley K, Beattie WS, Carroll J, Poonawala H, Ti L-K, Djaiani G (2025). Role of Cerebral Oximetry in Reducing Postoperative End-Organ Dysfunction After Major Non-Cardiac Surgery: A Randomized Controlled Trial. Clin. Pract..

[36] Lukaszewski M, Nelke K, Near-Infrared Spectroscopy (NIRS) in the Assessment of Cerebral Tissue Oxygenation (rSO2) (2024). Methodological Issues and Dilemmas. Anesthesia Research..

[37] Pedersen SS, Sørensen MK, Olsen MH (2024). Near-infrared spectroscopy to measure brain oxygenation: A comparison of measurements on the skin, skull and dura mater. Acta Anaesthesiol Scand.

[38] Tobias JD, Johnson GA, Rehman S, Fisher R, Caron N (2008). Cerebral oxygenation monitoring using near infrared spectroscopy during one-lung ventilation in adults. J Minim Access Surg.

[39] Leal-Noval SR, Arellano-Orden V, Muñoz-Gómez M (2017). Red Blood Cell Transfusion Guided by Near Infrared Spectroscopy in Neurocritically Ill Patients with Moderate or Severe Anemia: A Randomized, Controlled Trial. J Neurotrauma.

[40] Sen AN, Gopinath SP, Robertson CS (2016). Clinical application of near-infrared spectroscopy in patients with traumatic brain injury: a review of the progress of the field. Neurophotonics..

[41] Zhong W, Ji Z, Sun CA (2021). Review of Monitoring Methods for Cerebral Blood Oxygen Saturation. Healthcare.

[42] Moerman A, Wouters P (2010). Near-infrared spectroscopy (NIRS) monitoring in contemporary anesthesia and critical care. Acta Anaesthesiol Belg.

[43] Ruhatiya RS, Adukia SA, Manjunath RB, Maheshwarappa HM (2020). Current Status and Recommendations in Multimodal Neuromonitoring. Indian J Crit Care Med.

[44] Bögli SY, Beqiri E, Olakorede I (2025). Unlocking the potential of high-resolution multimodality neuromonitoring for traumatic brain injury management: lessons and insights from cases, events, and patterns. Crit Care..

[45] Frontera J, Ziai W, O'Phelan K (2015). Regional brain monitoring in the neurocritical care unit. Neurocrit Care.

[46] Magnussen AS, Olsen MH, Korshøj AR (2025). Multimodal neuromonitoring in the nordic countries: experiences and attitudes - a multi-institutional survey. Acta Neurochir (Wien)..

[47] Tas J, Czosnyka M, van der Horst ICC (2022). Cerebral multimodality monitoring in adult neurocritical care patients with acute brain injury: A narrative review. Front Physiol..

[48] Vitt JR, Loper NE, Mainali S (2023). Multimodal and autoregulation monitoring in the neurointensive care unit. Front Neurol..

[49] Sharma R, Tsikvadze M, Peel J, Howard L, Kapoor N, Freeman WD (2023). Multimodal monitoring: practical recommendations (dos and don'ts) in challenging situations and uncertainty. Front Neurol..

[50] Casault C, Couillard P, Kromm J, Rosenthal E, Kramer A, Brindley P (2022). Multimodal brain monitoring following traumatic brain injury: A primer for intensive care practitioners. J Intensive Care Soc.

[51] Robba C, Taccone F, Citerio G (2022). Monitoring cerebral oxygenation in acute brain-injured patients. Intensive Care Med.

[52] Veldeman M, Albanna W, Weiss M (2021). Invasive Multimodal Neuromonitoring in Aneurysmal Subarachnoid Hemorrhage: A Systematic Review. Stroke.

[53] Battaglini D, Santori G, Chandraptham K (2020). Neurological Complications and Noninvasive Multimodal Neuromonitoring in Critically Ill Mechanically Ventilated COVID-19 Patients. Front Neurol..

[54] Stocchetti N, Picetti E, Berardino M (2014). Clinical applications of intracranial pressure monitoring in traumatic brain injury: report of the Milan consensus conference. Acta Neurochir (Wien).

[55] Robba C, Pozzebon S, Moro B, Vincent JL, Creteur J, Taccone FS (2020). Multimodal non-invasive assessment of intracranial hypertension: an observational study. Crit Care..

[56] Le Roux P, Menon DK, Citerio G (2014). Consensus summary statement of the International Multidisciplinary Consensus Conference on Multimodality Monitoring in Neurocritical Care: a statement for healthcare professionals from the Neurocritical Care Society and the European Society of Intensive Care Medicine. Intensive Care Med.

[57] Foreman B, Kapinos G, Wainwright MS (2023). Practice Standards for the Use of Multimodality Neuromonitoring: A Delphi Consensus Process. Crit Care Med.

[58] Barrit S, Al Barajraji M, El Hadwe S (2024). Intracranial multimodal monitoring in neurocritical care (Neurocore-iMMM): an open, decentralized consensus. Crit Care..

